# The AIDS Pandemic: Impact on Science and Society

**DOI:** 10.3201/eid1111.050897

**Published:** 2005-11

**Authors:** Jeffrey L. Stephens

**Affiliations:** * Mercer University School of Medicine, Macon, Georgia, USA

**Keywords:** AIDS, book review, pandemic, society

As we enter the third decade of the AIDS pandemic, numerous texts explore the many aspects of AIDS and its consequences. Mayer and Pizer's premise is that AIDS has transformed many of the disciplines that it has touched. For the most part, this well-written volume supports their thesis. The authors, all established researchers, tackle many of the major issues, including virology, immunology, vaccines, microbicides, and sexually transmitted diseases, as well as the global impact of HIV/AIDS. Each chapter provides a well-referenced overview of its topic with many references as recent as 2003.

One of the real strengths of this book is a chapter on quantitative science that explores not only the history of HIV clinical trials, but also the design and importance of clinical trials in general. This chapter should be required reading for those considering clinical research in HIV. The chapters on Africa and Asia ably contrast the differences in these areas of highest prevalence. Another strength is the discussion of HIV in correctional facilities and the challenge of caring for this population, including their coexisting conditions and illicit drug use. Lastly, the discussion of the economics of AIDS is especially welcome in this era of efforts to increase access to drugs worldwide.

Overall, this book fills a valuable niche. A relatively concise text, it reviews many aspects of HIV with a focus on how each topic has evolved over the years. A few tables are small, but overall the diagrams and charts are clear and legible. This book would be of interest to infectious disease fellows, HIV caregivers, and those involved in public health and health policy. I heartily recommend this book and plan to keep it handy for future reference.

